# Developing an appropriate evolutionary baseline model for the study of SARS-CoV-2 patient samples

**DOI:** 10.1371/journal.ppat.1011265

**Published:** 2023-04-05

**Authors:** John W. Terbot, Parul Johri, Schuyler W. Liphardt, Vivak Soni, Susanne P. Pfeifer, Brandon S. Cooper, Jeffrey M. Good, Jeffrey D. Jensen

**Affiliations:** 1 University of Montana, Division of Biological Sciences, Missoula, Montana, United States of America; 2 Arizona State University, School of Life Sciences, Center for Evolution & Medicine, Tempe, Arizona, United States of America; University of Alberta, CANADA

## Abstract

Over the past 3 years, Severe Acute Respiratory Syndrome Coronavirus 2 (SARS-CoV-2) has spread through human populations in several waves, resulting in a global health crisis. In response, genomic surveillance efforts have proliferated in the hopes of tracking and anticipating the evolution of this virus, resulting in millions of patient isolates now being available in public databases. Yet, while there is a tremendous focus on identifying newly emerging adaptive viral variants, this quantification is far from trivial. Specifically, multiple co-occurring and interacting evolutionary processes are constantly in operation and must be jointly considered and modeled in order to perform accurate inference. We here outline critical individual components of such an evolutionary baseline model—mutation rates, recombination rates, the distribution of fitness effects, infection dynamics, and compartmentalization—and describe the current state of knowledge pertaining to the related parameters of each in SARS-CoV-2. We close with a series of recommendations for future clinical sampling, model construction, and statistical analysis.

## Introduction

**Fig 1:**
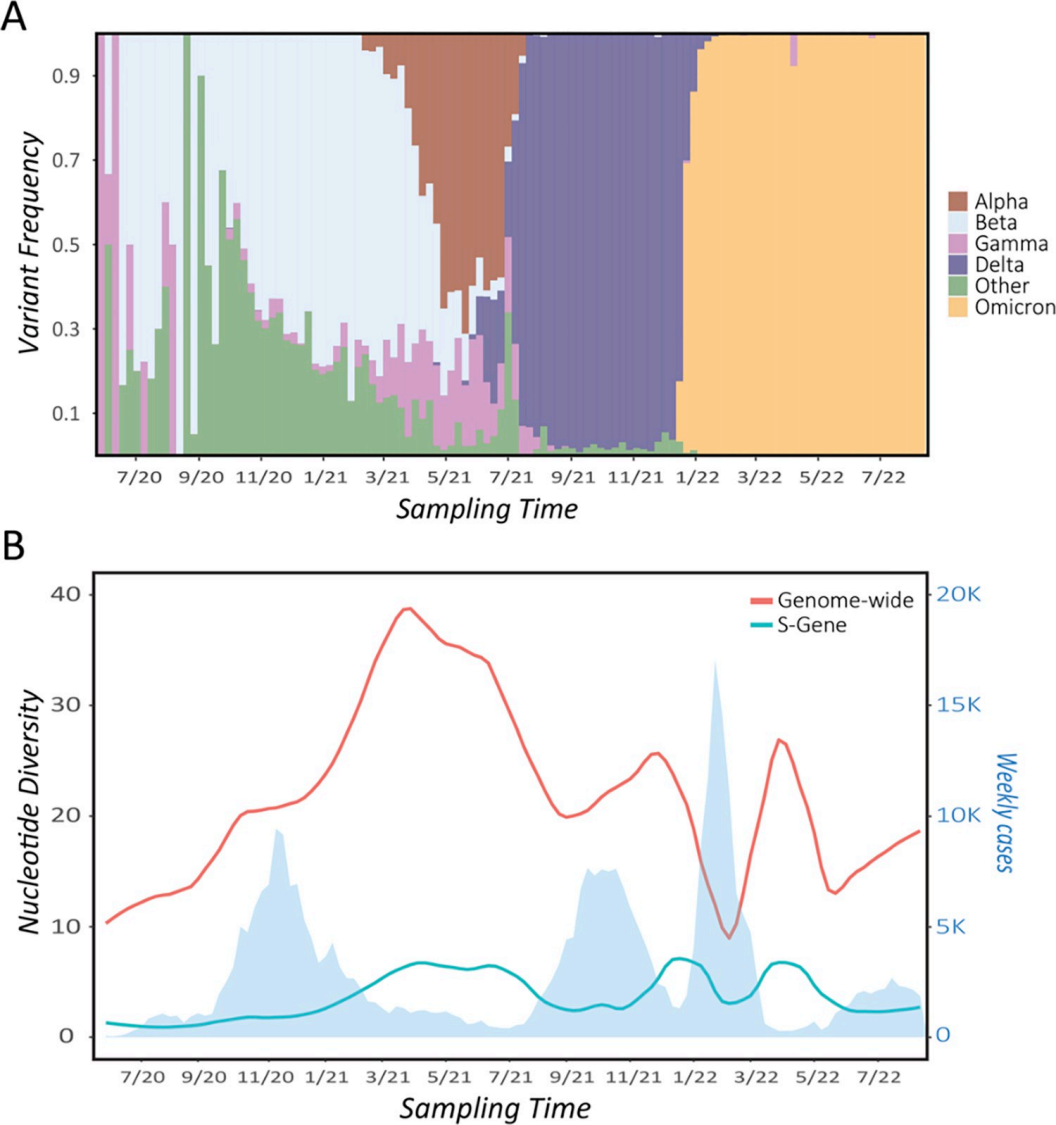
SARS-CoV-2 variant frequencies and genetic diversity through time, given for the state of Montana as illustration. **(A)** Frequency of major WHO-defined variants of concern (VOCs), binned by week of sampling as derived from GISAID metadata. **(B)** Average pairwise nucleotide differences between consensus sequence genomes isolated from patient samples genome-wide (orange line) and within the S-gene encoding the spike protein (blue line). As shown, local spread of major VOCs induced corresponding drops in overall genetic diversity across consensus sequences and within the S-gene. Note that while Beta dominated early in Montana, multiple variants cocirculated at appreciable frequency in 2020 to 2021, and the dominant observed strain differed by location. Consensus sequences were downloaded from GISAID (*n* = 21,799), binned by week, and aligned to the SARS-CoV-2 reference sequence using Nextalign. Diversity was calculated in R, using the package PopGenome. Local polynomial regression fitting (method loess) was used in the R package ggplot2 to model diversity through time with the formula y ~ x. Case data (given by the light blue shading) were downloaded from the CDC.

Current evidence suggests that Severe Acute Respiratory Syndrome Coronavirus 2 (SARS-CoV-2) emerged from Hubei Province, China, in late 2019 [1]. The virus has since spread through global human populations in several waves, infecting an estimated 612 million individuals and causing Coronavirus Disease 2019 (COVID-19), resulting in 6.55 million recorded deaths as of October 2022—though total estimates suggest 18.2 million fatalities in the first 2 years alone (January 2020 to December 2021; [2]). The situation has been exacerbated by the continued emergence of new variants of concern (VOCs) that continue to disrupt basic human health and activity (Fig 1), even after the development of vaccines that have significantly lessened COVID-19 severity. In particular, the Delta variant first identified in late 2020 in India was found to be highly transmissible; more recently, the emergence of Omicron—thought to have arisen from a long-term infection of an immunosuppressed individual [3]—led to even more rapid spread.

In response, global surveillance efforts have emerged to anticipate and track the ongoing evolution of this deadly virus using whole genome high-throughput sequencing. In fact, SARS-CoV-2 has rapidly become one of the most heavily resequenced genomes ever, with more than 13 million patient isolates to date. At the same time, several components of SARS-CoV-2 population biology and molecular evolution remain unresolved, potentially limiting both the accurate detection of emerging VOCs and the design of novel therapeutics (e.g., [4]). Here, we discuss the co-occurring evolutionary processes that must be jointly modeled to accurately study SARS-CoV-2 evolution. This includes outlining relevant SARS-CoV-2 mutation and recombination rate estimates, which govern the input of new genetic variation and the potential generation of novel combinations of variation. We additionally assess current information pertaining to the distribution of fitness effects, infection dynamics, and patterns of compartmentalization that, together with mutation and recombination, comprise a set of critical components of an evolutionary baseline model.

### A brief overview of key considerations

Fundamentally, when studying genome evolution, one should first begin with the realization that populations are simultaneously shaped by multiple evolutionary processes, including mutation, recombination, natural selection, and genetic drift. For this reason, it is often not feasible to accurately study any individual process in isolation without considering the input of all factors in shaping observed levels and patterns of genomic variation. For example, searching for genomic loci that have recently experienced positive selection—potentially leading to organismal adaptation—is particularly fraught as positive selection is expected to be rare and episodic relative to other constantly acting evolutionary processes (see reviews of [5,6]). Thus, the ability to successfully identify positively selected loci and differentiate this process from others is highly dependent on multiple underlying parameters. For example, it has previously been demonstrated that population bottlenecks, as well as high neutral mutational inputs, may be mistaken for the action of positive selection (e.g., [7–10]). For these reasons, the construction of an appropriate baseline model consisting of constantly acting evolutionary processes (e.g., mutation, genetic drift as modulated by population history, purifying selection, background selection, and so on) is critical to the successful detection and quantification of positive selection [11]. Importantly, as many of these underlying processes will be both heterogenous and estimated with uncertainty across the genome in question (e.g., mutation rates vary across a genome, and there is additionally measurement uncertainty in the underlying rate at any given site), the range of feasible parameter values needs to be modeled and considered within the context of this baseline.

All of these considerations also apply when the organism in question is a virus and the population concerned is contained within a patient. Human pathogens are often characterized by extreme mutational inputs, drastic population size changes relating to infection, reinfection, immune response, and clinical therapeutics, as well as often severe selective constraints owing to their coding-dense genomes [12]. Prior efforts have made significant progress in developing evolutionary baseline models for other common viruses (reviewed in [13]), and we here consider such model construction for the study of within-host SARS-CoV-2 populations. We outline critical individual components of a baseline model—mutation rates, recombination rates, the distribution of fitness effects, infection dynamics, and compartmentalization—and describe the current state of knowledge pertaining to the related parameters of each. Finally, we close with a series of recommendations for future clinical sampling, baseline model construction, and statistical analysis.

## Mutation rates

As the evolutionary source of new genetic variation, the first component of this baseline model necessarily involves the rate of input of new mutations. Numerous methods exist for estimating these rates, though the first complication that arises in comparing among studies is that some follow a convention of reporting rates per year or per day, while others per viral cycle. To compare among such estimates for SARS-CoV-2, we consider the maximum number of sequential viral cycles thought to occur in a single year for use as a conversion factor. Specifically, based on cycle times in SARS-CoV-1, the time required to enter a host cell is approximately 10 minutes and the eclipse period of the virus (i.e., the time between host cell entry and the generation of new infectious particles) is approximately 10 hours [14], leading to a conversion factor of 861.64 viral cycles/year (i.e., the number of 610-minute cycles in a calendar year). While such a conversion is necessary in order to compare between existing estimates, variation in these entry and eclipse times would naturally result in a modified scaling factor.

Molecular clock-based substitution rates previously observed in other betacoronaviruses provide the first insight into relevant mutation rates (Table 1); however, such clock-based rates ought to be regarded as more closely approximating the rate of neutral mutations, rather than the rate of all mutations. Using such a phylogenetic approach, this rate has been estimated at 3.5 × 10^−6^ mutations/nucleotide (nt)/cycle for murine hepatitis virus (MHV) [15,16] and at 0.80 to 2.87 × 10^−3^ mutations/nt/year for SARS-CoV-1 [17], corresponding to 0.93 to 3.33 × 10^−6^ mutations/nt/cycle following our above conversion. Given the abundance of sequence data in public databases (e.g., [18,19]), comparable rate estimates have also recently been reported for SARS-CoV-2. For example, using SARS-CoV-2 sequences generated prior to April 2020 and estimating genetic distance to closely related bat and pangolin coronaviruses, an initial clock-based estimate was inferred to be 1.87 × 10^−6^ mutations/nt/day (approximately 7.92 × 10^−7^ mutations/nt/cycle) [20]. Other estimates have focused on rates of divergence over time within humans. An early analysis based on 73 genomes collected between December 2019 and February 2020 estimated a mutation rate of 1.19 to 1.31 × 10^−3^ mutations/nt/year [21], or 1.38 to 1.52 × 10^−6^ mutations/nt/cycle. A subsequent study based on 137 genomes estimated a rate of 2.4 × 10^−3^ mutations/nt/year (95% CI: 1.5 to 3.3 × 10^−3^ mutations/nt/year; [22]), or 2.79 × 10^−6^ mutations/nt/cycle (95% CI: 1.74 to 3.83 × 10^−6^ mutations/nt/cycle)—a rate similar to other estimates from the same period using similar methods [23].

**Table 1:** Estimated mutation rates in SARS-CoV-2, and the related CoV-1 and MHV.

Virus	Source	Date	Original Unit	Estimated Rate / Cycle	SEM	Citation
MHV	in vitro	June 2004	mut/nt/cycle	3.5 × 10^−6^ mut/nt/cycle	n/a	[15,16]
SARS-CoV-1	divergence	June 2004	mut/nt/yr*	0.93–2.76 × 10^−6^ mut/nt/cycle	n/a	[17]
SARS-CoV-2	divergence	February 2021	mut/nt/day*	0.79 × 10^−6^ mut/nt/cycle	n/a	[20]
SARS-CoV-2	divergence	February 2020	mut/nt/yr*	1.38 × 10^−6^ mut/nt/cycle	0.35**	[21]
SARS-CoV-2	divergence	February 2020	mut/nt/yr*	1.52 × 10^−6^ mut/nt/cycle	0.36**	[21]
SARS-CoV-2	divergence	June 2020	mut/nt/yr*	2.79 × 10^−6^ mut/nt/cycle	0.53**	[22]
SARS-CoV-2	divergence	February 2021	mut/nt/yr*	1.71 × 10^−6^ mut/nt/cycle	0.23**	[23]
SARS-CoV-2	divergence	August 2022	mut/nt/yr*	0.72 × 10^−6^ mut/nt/cycle	n/a	[26]
SARS-CoV-2	in vitro	March 2022	mut/nt/cycle	1.3 × 10^−6^ mut/nt/cycle	0.1	[24]

*Evolutionary rates reported as per year or per day mutation rates were converted to per cycle mutation rate using the approximation of 861.64 viral cycles per year.

**SEMs (standard errors of the mean) estimated based on reported 95% HPD (highest posterior density) intervals.

An alternative approach for estimating mutation rates instead relies on serial sampling within an experimental setting or from individual patients. This serial sampling approach has the advantage of not suffering the data reduction associated with comparisons between consensus sequences (see Data consideration section below) and should capture a wider (but still limited) range of newly emerging mutations. For example, sampling over 15 days from lines of African green monkey (*Chlorocebus aethiops*) kidney cell cultures inoculated with a clinical isolate led to an observed rate between 2.9 and 3.7 × 10^−6^ mutations/nt/cycle across the genome [24]. However, after excluding the nsp3, nsp6, and S genes—which showed evidence of selection potentially biasing the mutation accumulation-based inference—estimates were reduced to 1.3 ± 0.2 × 10^−6^ mutations/nt/cycle (and see [24] for a more mechanistic discussion of these contributing variants).

In addition to the overall mutation rate, consideration needs to be given to the extent to which mutation rates may vary across the SARS-CoV-2 genome. The causes of any apparent mutation rate heterogeneity also require attention given that differential selection across genomic regions may be responsible for these patterns rather than a variable underlying rate itself. For example, based on comparisons between 4-fold degenerate sites relative to other sites in the genome, it was estimated that the genuine mutation rate may be 49% to 67% higher than that estimated based on common segregating variants [25]. Additionally, a continuous reduction in the nonsynonymous (but not synonymous) mutation rate has been observed throughout the pandemic [26]—a trend likely indicating changes in selection pressures rather than mutation rates. For this reason, a consideration of the proportion of new mutations that are deleterious, neutral and beneficial (see Distributions of fitness effects section below) will be important for understanding the total rate of new mutation and distinguishing it from rates estimated from segregating variation.

In this regard, it is important to also consider the genomic composition of SARS-CoV-2, which is approximately 29.8 kilobases (kb) and contains 25 to 30 distinct regions that encode gene products. As expected, genic regions generally appear highly constrained, while a subset of genes, including the spike gene (S), appear to evolve comparatively rapidly. Mechanistically, RNA-dependent RNA polymerases are known to be error-prone [4,27,28], which in coronaviruses is partly compensated by a 3′ to 5′ proofreading exonuclease, nsp14 [29]. The impact of this sort of “evolutionary layering” remains in need of further study [4,30]. Notably, mutations within the replicative machinery (nsp12 and nsp14) can directly lead to increases in mutation rates. For example, using inoculated kidney cells from the African green monkey, mutator lines have been observed to develop which accumulated mutations at roughly an order of magnitude greater rate [24]. These mutator lines were found to have several nonsynonymous mutations in their replication machinery unique to those lineages (8 in nsp12, and 9 within nsp14). Such observations have in fact led to the suggestion that drug-induced mutational meltdown may itself represent a viable patient-treatment strategy (e.g., [4,31]), as has previously been suggested in other viruses (e.g., [32]).

## Recombination rates

Though mutation is the source of new genetic variation, recombination is an important process for generating novel combinations of variation. Additionally, by disrupting selective interference between and among mutations (i.e., Hill–Robertson interference; [33,34]), recombination may improve the efficacy of both positive and purifying selection.

Recombination has been observed in many viruses, including coronaviruses [4,35–38]. Indeed, recombination events between different coronavirus lineages are thought to have been essential for the evolution of SARS-CoV-2. Specifically, several critical recombinant regions were identified in the genome; 3 within the spike protein and 1 associated with each of the RNA-dependent RNA polymerase [39,40]. An early report using a sequence similarity search of a local database of SARS-CoV-2 samples and other coronaviruses concluded that there were sequences derived from coronaviruses of bats and, potentially, pangolins [20]. Phylogenetic approaches using sliding window analyses have found a similar mosaic origin for SARS-CoV-2 [41–44]. Moreover, recombination between SARS-CoV-2 lineages was detected as early as April 2020, using analyses based on linkage disequilibrium [20]. A subsequent tree-based analysis constructed from 1.6 million sequences identified 606 putative recombination events, suggesting that 2.7% of circulating strains likely had a recombinant origin [45]. Importantly, the accurate computational estimation of these rates requires the analysis of within-host polymorphism data, rather than consensus sequence data as is common, suggesting that this frequency may be underestimated [46].

A major limiting factor for recombination detection is that it requires a coinfection by 2 or more strains [47,48]. Moreover, this coinfection needs to not only occur within a single host, but the viral strains also need to infect the same host cell. Coinfections may be relatively rare—estimated to occur in 0.18% to 0.61% of samples [48–50]—though this rate would be expected to increase alongside increases in viral occurrence [47]. Additionally, within-host dynamics will impact the duration of the infection—the longer an infection persists, the greater the opportunity for a secondary infection to be acquired [48].

The analysis of recombination breakpoints has identified possible hotspots within the SARS-CoV-2 genome [51], suggesting that, as with mutation, rate heterogeneity across the genome may be important. These hotspots are often associated with transcription regulatory sequences (TRSs) found nearby various open reading frames (ORFs) and are associated with the template switching process, which produces sgRNA. Notably, recombination junctions that were associated with TRSs were less likely to produce defective viral genomes [52]. Microhomologies of 2 to 7 nucleotides between recombination junctions of SARS-CoV-2 and MHV were also identified, potentially suggesting some level of conservation [52]. Finally, the recombination rate may also be related to the proofreading enzyme found in coronaviruses. When this gene was knocked out in lines of MHV, there was a significant reduction in recombination rates in addition to altered recombination patterns [52].

The estimated rates of recombination for coronaviruses, including SARS-CoV-2, occur within a considerable range. Work in MHV estimated the recombination rate of that virus to be roughly 1 × 10^−5^ between consecutive sites [53]. A recent study in SARS-CoV-2, seeking to detect recombination using a parsimony approach [54], had a sensitivity that allowed recombination detection given at least a minimal rate of 1 × 10^−6^ recombination events/site/cycle [55]. Recombination was successfully detected by this method, providing a possible minimum for the recombination rate. An alternate approach using a Markov chain Monte Carlo method to infer recombination networks under a template-switching model of recombination [56] inferred a rate of 2 × 10^−6^ events/site/year. Using the same conversion factor used previously for mutation rates, this would suggest an approximate recombination rate of 2.32 × 10^−9^ events/site/cycle—substantially below the detection floor of the parsimony-based method referenced above, likely owing to considerations related to coinfection rates. Thus, these estimates, combined with the uncertain rates of coinfection, suggest considerable ambiguity in this parameter space. However, current evidence would imply recombination rates in the range of 1 × 10^−5^ to 1 × 10^−6^ events/site/cycle in coinfections (i.e., pertaining to <1% of total infections).

## Distributions of fitness effects

The SARS-CoV-2 genome, albeit sizeable for an RNA virus, is nonetheless relatively small and highly compact, with >95% of the genome thought to be functionally significant [57,58]. Given this paucity of nonfunctional sequence, and that many ORFs are overlapping, it will likely be challenging to identify neutrally evolving sites in the SARS-CoV-2 genome in sufficient numbers to allow for common neutrality-based inference approaches. That said, synonymous sites are possible candidates, with a recent study [26] suggesting that the rate of synonymous substitution appears to be roughly constant over time, indicating that synonymous sites in SARS-CoV-2 may be evolving nearly neutrally.

However, while the frequency distribution of neutral alleles is shaped by demographic events (e.g., the population bottleneck and subsequent growth associated with infecting a host)—allowing us to make inferences about population sizes [59]—it is also impacted by the linked effects of selection acting on nearby functionally important sites ([60,61]; and see review of [6]). For this reason, it has been shown to be necessary to jointly infer the distribution of fitness effects (DFE) together with population history when analyzing coding-dense genomes [62–64]. In other words, while neutral mutations surely exist, fully degenerate sites that are unlinked to constrained sites may not.

**Fig 2:**
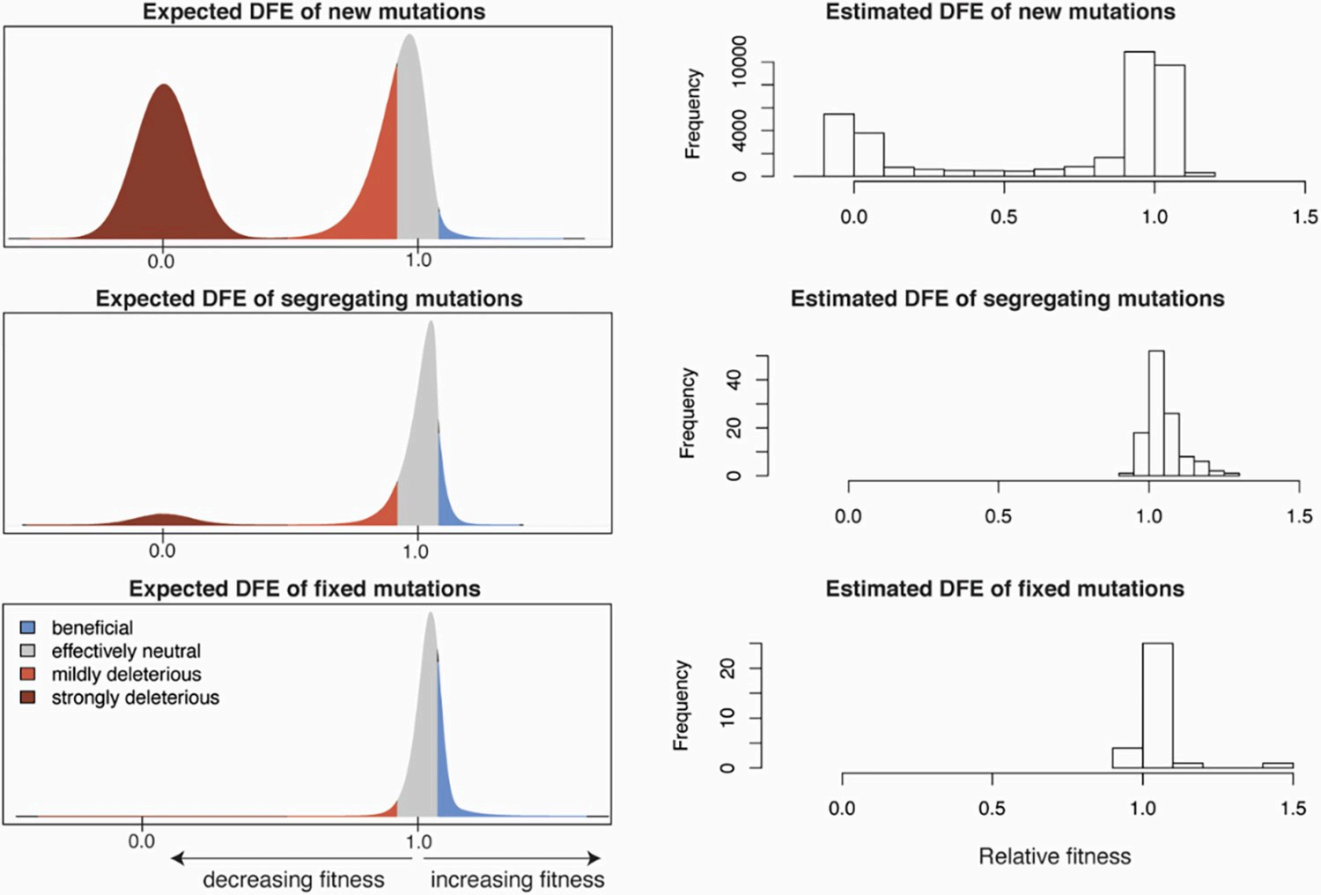
The expected and estimated distributions of fitness effects (DFE) of mutations. Left panels: a hypothetical expected DFE of new, segregating, and fixed mutations, reflecting the effects of selection at each stage. Effectively neutral mutations are shown in gray, beneficial mutations in blue, and deleterious mutation in shades of red. Right panels: the DFE of new, segregating, and fixed amino acid variants in SARS-CoV-2 as recently estimated by Flynn and colleagues [131], Kepler and colleagues [65], and Obermeyer and colleagues [132], respectively. From Flynn and colleagues, the normalized functional scores from 2 sets of biological replicates were pooled together. From Kepler and colleagues, the relative fitness of all single mutations from pre- and post-2020 studies were pooled together. From Obermeyer and colleagues, the DFE of fixed mutations was approximated by using 31 high-frequency variants (defined as those present in more than 100 lineages). Importantly, observed genomic variation will depend heavily on the underlying heterogeneity in both mutation rates and DFEs across the genome, among other factors [133].

There has been some work to date to characterize the DFE of observed mutations sampled from SARS-CoV-2 patients, using a variety of approaches. Accounting for the background rate of growth/transmission for each geographic region in the United States separately, Kepler and colleagues [65] used a maximum likelihood phylodynamic method to infer the fitness effects of segregating amino acid variants from 88,000 viral genomes. Under this framework, the fitness of each lineage on the tree was estimated using a birth–death process (such that fitness of the parent and child branch was correlated). As expected, most segregating variants were found to be effectively neutral, with a small minority being mildly deleterious and approximately 20% being putatively beneficial (Fig 2). More specifically, prior to 2020, approximately 14% (7 out of 51) of all amino acid variants segregating were inferred to be significantly beneficial with an average fitness advantage of 1.15 relative to the wild type. Strongly beneficial mutations are expected to fix rapidly in populations, and strongly deleterious mutations are likewise expected to be purged quickly (conditional on fixation and loss, respectively); therefore, most segregating variants sampled at any given time are likely to be neutral or weakly selected, consistent with this inferred DFE of segregating variants (see Fig 2).

More generally, multiple studies (e.g., [66–68]) have found significantly fewer segregating alleles at nonsynonymous relative to synonymous sites, likely reflecting the effects of purifying selection acting on functional changes. For example, Neher [26] evaluated patterns of segregating alleles and found that 15% to 20% of first and second codon positions exhibit no observed variation. This is consistent with previous random mutagenesis studies in other coding-dense RNA viruses, which have suggested that 20% to 40% of all new mutations are likely strongly deleterious [69,70]. Interestingly, some ORFs also appear to be experiencing mild selective constraints, as evidenced by comparison with synonymous sites [26], further suggesting that an appreciable class of mutations may also be weakly deleterious.

Thus, while considerable study is required to further characterize within-patient DFEs—particularly accounting for the simultaneous contributions of population size change, direct selection, and selection at linked sites [71]—current studies have provided first glimpses into the relative occupancy of different DFE classes. Furthermore, phylogenetic methods utilized to date neglect the effects of recombination, further highlighting the value of future applications of population genetic-based inference approaches. With regard to baseline model construction specifically, the uncertainty in the underlying DFE may be evaluated by modeling the effects of different possible densities in the 2 modes of the DFE, utilizing a range of generalized densities (e.g., [72]).

## Infection dynamics

**Fig 3:**
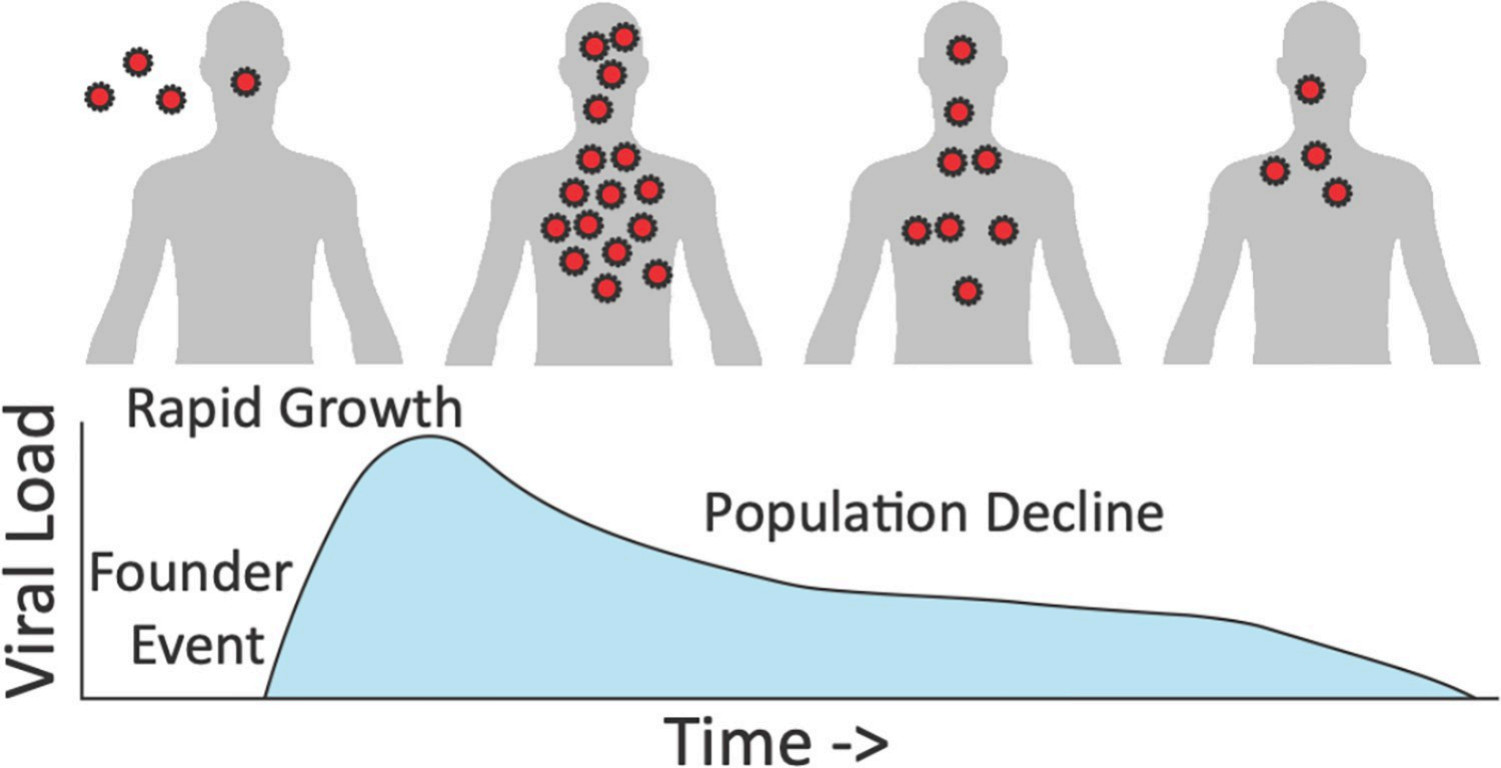
A schematic of a simple intra-host demographic model potentially underlying infection dynamics. At the time of infection, the virus population will initially be characterized by a population bottleneck associated with the founder event. A successful infection will next be characterized by rapid population growth associated with high viral loads, and reducing sizes and loads as the patient begins to clear the infection. The details of this infection history will greatly shape the observed levels and patterns of intra-host diversity. Viral load schematic modified from [134].

Within-host population dynamics are important determinants of levels and patterns of variation, as well as of potential selective outcomes (Fig 3). Generally, a population bottleneck tends to be associated with initial patient infection, followed by rapid population growth (see review of [13]). The size of the transmission bottleneck is an important factor in determining how much within-host genetic variation is initially present, on which selection may act [73]. A narrow transmission bottleneck can result in a severe loss of genetic variation (known as a founder effect), with low-frequency within-host variants being stochastically lost from the population, largely regardless of their fitness effects. Conversely, if the transmission bottleneck is wide, then there may be numerous viral particles founding the initial infection, increasing the chance that beneficial variants are maintained.

Several studies [66,74–80] have attempted to infer the severity of the SARS-CoV-2 infection bottleneck using the beta-binomial method [81], though estimates have ranged considerably, from 1 to 8 infecting particles [66] to more than 1,000 [74]. It has also been shown that the frequency threshold for detecting variants impacts bottleneck estimates [77–79], with an increased frequency threshold resulting in decreased bottleneck size estimates. There are fewer studies that have considered the increase in population size following the transmission bottleneck. Du and colleagues [81] performed a systematic review of viral dynamic parameters used in within-host models, estimating a mean viral load at symptom onset of 4.78 (95% CI: 2.93, 6.62) log(copies/ml) across 3 models, compared with a mean viral load at point of infection of −1.00 (95% CI: −0.94, −0.05) log(copies/ml), which puts a wide range on the respective rate of postinfection expansion. Wang and colleagues [82] compared genetic diversity in intra-host populations from the respiratory and gastrointestinal tract, finding considerably less diversity in the former than in the latter. One possible intra-host migration route is from the respiratory tract to the gastrointestinal epithelia, suggesting that this reduction in diversity in the respiratory tract may be due to the infection bottleneck, followed by rapid recovery in the gastrointestinal tract (though see the below section on Compartmentalization as well).

The considerable variance in these estimates suggest that there is still important work to be done in inferring a demographic infection model and its underlying parameters for SARS-CoV-2. Such inference is inherently challenging due to the impact of selection on biasing demographic inference as discussed above (and see [83,84]), and indeed due to the impact of demography on biasing selection inference [62,85]. To account for this circular problem, methods that jointly infer demography and the DFE will be critical. Although numerous joint estimation approaches for demographic inference have been developed, the most appropriate approach will be dependent on the context in which it is applied (considerations of which are reviewed in [11]). Neutral demographic estimators require sufficiently large nonfunctional regions and high rates of recombination, such that assumptions of strict neutrality hold [86–89]. Specifically, these criteria ensure that variants can be chosen that are not experiencing background selection. For example, Renzette and colleagues [90] utilized a neutral demographic inference approach (*dadi*; [86]) to build and parameterize infection models in human cytomegalovirus (HCMV) (and see [91,92]). It is notable, however, that the HCMV genome is 236 kb in size—among the largest human viral genomes [93]—and has large noncoding regions [94]. By contrast, the smaller, largely functional SARS-CoV-2 genome [95,96] likely prohibits such neutral inference. For these reasons, recently proposed approximate Bayesian computation (ABC) approaches to estimate demography while accounting for background selection effects will likely be the most fruitful path forward [62].

Of additional importance in considering viral infection dynamics is the notion of progeny skew—i.e., the viral replication dynamics. A majority of population genetic inference approaches assume small progeny distributions—an assumption that is likely violated in many pathogens (see reviews of [12,97]). Helpfully, recent inference approaches have relaxed this assumption, demonstrating an ability to coestimate parameters related to the biology of progeny skew together with those of demographic and selective histories (e.g., [98,99]). Moreover, progeny skew has been incorporated into the joint ABC inference scheme noted above, demonstrating an ability to tailor such inference specifically to viral populations [64,100], and, importantly, to avoid the misinference resulting from a neglect of this consideration.

## Compartmentalization

Related to the population dynamics discussed above, which were largely concerned with population size changes associated with infection, the compartmentalization of viral populations within an infected individual (i.e., within-host population structure)—either across tissue types or regions of a single tissue—can also play a key role in the intra- and inter-host evolutionary dynamics of a virus. For example, HIV is known to spread throughout the body during early stages of infection leading to distinct viral populations localized to certain organs or systems, resulting in populations evolving independently under unique evolutionary pressures [101]. Influenza A virus has been shown to compartmentalize within different lobes of the lungs resulting in genetically distinct populations, each with distinct evolutionary histories. Similarly, HCMV has been shown to have strong compartmentalization effects, with the plasma population facilitating a certain degree of gene flow between compartments [90,102–105].

It has been apparent since early in the pandemic that SARS-CoV-2 also demonstrates a certain level of intra-host compartmentalization, with the identification of unique variants not shared between the upper and lower respiratory tracts in patients presenting severe disease [106,107]. This is perhaps not surprising, given that previous studies have observed compartmentalization between upper and lower respiratory tracts in both SARS-CoV-1 and MERS [108,109]. Recent sequencing efforts across a larger number of samples, and to a greater coverage across the viral genome, have recapitulated this pattern of differentiation between the upper and lower respiratory tracts in SARS-CoV-2 [110]. Intra-host single nucleotide variants not shared between blood and upper respiratory tract samples were also recovered from a single immunocompromised patient in France [111]. SARS-CoV-2 compartmentalization across different organs has been studied to a lesser degree; however, organ-specific variants, including the observation of VOC mutations outside of lung tissue, have been reported [112]. For example, compartmentalization has been observed between the respiratory tract and the gastrointestinal tract, with unique variants recovered from different samples [82].

Early evidence suggests that the evolutionary dynamics within compartments may be at least initially shaped by stochastic processes for SARS-CoV-2—such as founder events resulting in genetically distinct compartmental populations—similar to patterns seen in other viruses [110,113]. Further, several recent studies have demonstrated that the ability of the Omicron variant to replicate in the lungs is severely reduced compared to that of the nasal tract, potentially suggesting compartmentalization effects in this regard as well [114,115]. As such, this within-host structuring owing to compartmentalization represents another important component of any underlying evolutionary baseline model.

## Data considerations

**Fig 4:**
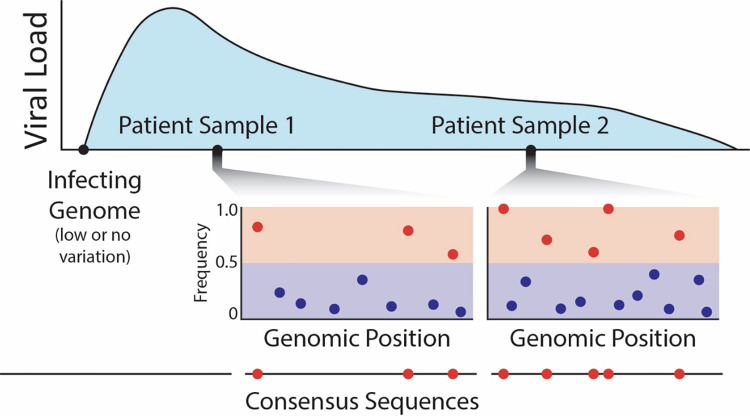
An example of clinical sampling of a patient over the course of an infection, demonstrating how a consensus sequence-based summary neglects the great majority of intra-host variants (which are expected to primarily segregate at low frequencies [shown as blue circles]). This ascertainment of high-frequency intra-host variants (shown as red circles) for subsequent inter-host comparison thus represents an unfortunate and unnecessary loss of information.

Apart from the considerations of contributing evolutionary processes discussed in the sections above, it is additionally important to consider the types of data needed and available to perform such evolutionary inference. As of October 2022, there were over 6.3 million SARS-CoV-2 genomes deposited in NCBI, and over 13.4 million genomes deposited in GISAID [116]. Consensus sequences (i.e., single-sequence representations of a patient’s viral population) produced from individual samples comprise most of these data, and these sequences serve as the primary unit for most genetic analyses of SARS-CoV-2 lineage variation and evolution. While consensus sequences provide the opportunity to carry out analyses concerning inter-host viral variation across millions of infected patients, they completely obscure intra-host variants segregating within patients (Fig 4). This preventable loss of information precludes analysis of variation within hosts and can lead to incorrect evolutionary inference regarding the relative contributions of respective evolutionary processes during viral evolution [13,104]. More fundamentally, consensus-level variation is simply a summarized by-product of the multiple contributing evolutionary processes acting within hosts. At a minimum, we emphasize that depositing the raw reads from which consensus sequences were constructed would be a helpful step in allowing subsequent researchers to examine individual host-level variation. Indeed, as is already standard in other scientific fields, depositing raw sequencing data to public repositories should be adopted as a best practice for viral genome surveillance in order to assure scientific transparency and reproducibility.

Relatedly, how and where SARS-CoV-2 genomes are deposited can create barriers to carrying out analyses of intra-host variation. For example, researchers may submit to NCBI and / or GISAID, and submissions may or may not include both raw reads and consensus sequences. This also makes it challenging to reliably cross-reference samples and underlying data between databases. Still, as with the yellow fever virus [117], influenza [118], norovirus [119], Ebola [120], and other RNA viruses [121,122], analyses of available raw-read data have revealed significant intra-host SARS-CoV-2 variation [82,110,123]. Notably, individual patients who share consensus-level variation may generally have very different intra-host variation [82], highlighting how analyses focused only on inter-host variation may fail to capture the vast majority of relevant SARS-CoV-2 variation and evolution.

Relatedly, evolutionary studies of intra-host and inter-host variation have historically produced conflicting results, whereby intra-host variation is much greater than inter-host variation. For example, analysis of HCMV populations within hosts has reported per-site nucleotide diversity values that that differ by an order of magnitude from estimates of between host per-site nucleotide diversity [104,124,125]. These analyses indicate that approximately 68% of HCMV genomic sites are polymorphic within hosts, while only 12% of sites are segregating among hosts. Some researchers have interpreted these patterns as reflecting that most viral polymorphisms sampled within hosts are strongly deleterious [126,127]. However, population genetic analyses have demonstrated that these observed differences within and among hosts are consistent with most observed viral polymorphisms being nearly neutral with regard to fitness—indeed, standard population genetic models seem to fully capture both observed intra-host and inter-host HCMV variation [91,104,125].

Applying similar approaches to SARS-CoV-2 will be crucial to better understand its evolution and global spread, but this first requires the development of a baseline model as discussed herein. This development, in turn, will be aided by sampling and sequencing SARS-CoV-2 populations from single patients at multiple time points (Fig 4). While still relatively rare, several studies have recently produced time-sampled whole-genome SARS-CoV-2 data [66,82,110,128]. For example, one study sampled 41 patients at 2 time points, collected on average 6 days apart, and observed both generation and loss of intra-host variation during this period [66]. Most importantly, additional time-series data will allow for the intra-host description of temporal changes in allele frequencies providing greatly improved resolution on the parameters underlying selection, population size change, and population structure [129,130], and will further provide the data necessary to perform needed inference to better quantify underlying rates of mutation and recombination.

## Concluding thoughts

Though this virus currently represents a unique global threat, it is also simply an organism like any other that can be studied using basic population genetic principles. While the many considerations here described may appear rather complex, it is our hope that these recommendations together with this compendium of recently obtained estimates will prove useful in future efforts to better illuminate the within-patient evolutionary dynamics of SARS-CoV-2. Without this framework to define hypotheses and accurately quantify contributing evolutionary processes, epidemiological and genetic data describing spatial and temporal variations in disease incidence and mutational frequencies will remain merely descriptive and thus will alone be unable to broach the key evolutionary questions of greatest relevance to public health.
